# Phase-Dependent Response to Afferent Stimulation During Fictive Locomotion: A Computational Modeling Study

**DOI:** 10.3389/fnins.2019.01288

**Published:** 2019-11-29

**Authors:** Soichiro Fujiki, Shinya Aoi, Kazuo Tsuchiya, Simon M. Danner, Ilya A. Rybak, Dai Yanagihara

**Affiliations:** ^1^Department of Physiology and Biological Information, Dokkyo Medical University School of Medicine, Mibu, Japan; ^2^Department of Aeronautics and Astronautics, Graduate School of Engineering, Kyoto University, Kyoto, Japan; ^3^Department of Neurobiology and Anatomy, Drexel University College of Medicine, Philadelphia, PA, United States; ^4^Department of Life Sciences, Graduate School of Arts and Sciences, The University of Tokyo, Tokyo, Japan

**Keywords:** central pattern generator, half-center CPG, afferent control of CPG, phase-dependent response, dynamic structure

## Abstract

Central pattern generators (CPGs) in the spinal cord generate rhythmic neural activity and control locomotion in vertebrates. These CPGs operate under the control of sensory feedback that affects the generated locomotor pattern and adapt it to the animal's biomechanics and environment. Studies of the effects of afferent stimulation on fictive locomotion in immobilized cats have shown that brief stimulation of peripheral nerves can reset the ongoing locomotor rhythm. Depending on the phase of stimulation and the stimulated nerve, the applied stimulation can either shorten or prolong the current locomotor phase and the locomotor cycle. Here, we used a mathematical model of a half-center CPG to investigate the phase-dependent effects of brief stimulation applied to CPG on the CPG-generated locomotor oscillations. The CPG in the model consisted of two half-centers mutually inhibiting each other. The rhythmic activity in each half-center was based on a slowly inactivating, persistent sodium current. Brief stimulation was applied to CPG half-centers in different phases of the locomotor cycle to produce phase-dependent changes in CPG activity. The model reproduced several results from experiments on the effect of afferent stimulation of fictive locomotion in cats. The mechanisms of locomotor rhythm resetting under different conditions were analyzed using dynamic systems theory methods.

## Introduction

The mammalian spinal cord contains neuronal circuitry that can generate a basic locomotor rhythm and produce the alternating flexor and extensor motoneuron activities underlying locomotion. Although this locomotor central pattern generator (CPG) can operate in the absence of sensory feedback (reviewed by Grillner, 1981; Rossignol, 1996; Orlovsky et al., 1999; Rossignol et al., 2006), afferent feedback plays a crucial role in adjusting the locomotor pattern to the motor task, the environment, and the biomechanical characteristics of the limbs and body (e.g., Pearson, 2004; Rossignol et al., 2006). Continuous electrical stimulation of the midbrain locomotor region in an immobilized decerebrate cat produces “fictive locomotion” consisting of rhythmic alternating activation of flexor and extensor motoneurons similar to that occurring during normal locomotion in an intact animal (see Rossignol, 1996). To investigate the effects of afferent inputs on the locomotor pattern generated by the CPG and step cycle timing, researchers often use the fictive locomotor preparations while applying stimulation to flexor or extensor sensory afferents (e.g., Guertin et al., 1995; Perreault et al., 1995; McCrea, 2001; Stecina et al., 2005). These studies revealed that in many cases, afferent stimulation can delay or accelerate the phase transition within the ongoing step cycle with or without changing the timing of the subsequent step cycles (Rybak et al., 2006b; McCrea and Rybak, 2007).

Although the anatomical structure of the CPG circuit remains unclear, the use of relatively simple mathematical models of CPGs allows the study of the general effects of afferent stimulation on CPG operation from a dynamic viewpoint. In particular, half-center type CPG models were previously used to reproduce some effects of sensory afferent stimulation on fictive locomotor pattern in cats (Rybak et al., 2006b).

The goal of the present study was to further investigate the mechanism for the phase-dependent response of the locomotor pattern during fictive locomotion using a simplified half-center CPG model. Specifically, we applied stimulation to the CPG model in different phases of the locomotor cycle and examined how the temporal activity of the CPG changed. The use of a relatively simple CPG model allowed us to apply the dynamic system methods and perform mathematical analysis to fully characterize the phase-dependent responses of the CPG to applied stimulation.

## Methods

### Model

**Figure 1:**
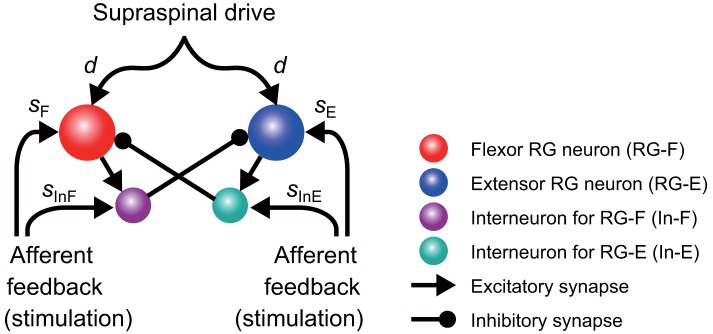
Model schematic of the rhythm generator (RG) network and afferent inputs. The RG network is composed of flexor (RG-F) and extensor (RG-E) centers inhibiting each other via inhibitory interneurons In-F and In-E, respectively. The supraspinal drive provides excitation to the RG-F and RG-E neurons defining the frequency of oscillations. Sensory afferents can synaptically excite both RG neurons and inhibitory interneurons.

It has been suggested that the rhythmic pattern of the CPG activity is determined in the rhythm generator (RG) network of the CPG (Rybak et al., 2006a,b). In the present study, the model (Figure 1) consisted of two neuron populations representing RG centers (flexor RG-F and extensor RG-E) and two populations of inhibitory interneurons (In-F, In-E), providing mutual inhibition between the flexor and extensor centers. Each population was described as an activity-based (non-spiking) neuron model (Ermentrout, 1994; Markin et al., 2010; Molkov et al., 2015; Danner et al., 2016, 2017). The state of each neuron was characterized by the membrane potential *V*_*i*_ (*i* = F, E, IF, IE), where the indexes F and E are used for the RG-F and RG-E neurons, respectively, and the indexes IF and IE are used for the In-F and In-E neurons, respectively. The RG-F and RG-E neurons incorporated a persistent (slowly inactivating) sodium current that defined intrinsic rhythmogenic properties of these neurons. The intrinsic oscillation in each RG neuron depended on the variable *h*_*i*_ (*i* = F, E) that defined slow inactivation of the persistent sodium channels. Each RG center could produce rhythmic activities; however, if uncoupled, the extensor center was in the tonic regime due to a supraspinal drive and produced sustained activity. Rhythmic oscillations of the RG were defined by the flexor centers, which provided rhythmic inhibition of the extensor center through In-F. The supraspinal drive to the flexor center determined the oscillation frequency. Synaptic interactions between all neurons in the model are shown in Figure 1. For the state variable of this model, we used $V={\left[V_{\text{F}},V_{\text{E}},V_{\text{IF}},V_{\text{IE}}\right]}^{T}$ and $h={\left[h_{\text{F}},h_{\text{E}}\;\right]}^{T}$.

The dynamics of the membrane potential *V*_*i*_ of the RG neurons (*i* = F, E) and the interneurons (*i* = IF, IE) is described as

(1) $$\begin{array}{l} {\text{C}\dot{V}}_{i}=\{\begin{array}{c} -I_{\text{NaP}}\left(V_{\text{i}},h_{\text{i}}\right)-I_{\text{Leak}}\left(V_{\text{i}}\right)-I_{\text{SynE}}^{i}\left(\;V\right)-I_{\text{SynI}}^{i}\left(\;V\right)\;i=\text{E},\text{F }\\ -I_{\text{Leak}}\left(V_{\text{i}}\right)-I_{\text{SynE}}^{i}\left(V\right)-I_{\text{SynI}}^{i}\left(\;V\right)\;i=\text{IF },\text{IE} \end{array} \end{array}$$

where *C* is the membrane capacitance, *I*_NaP_ is the persistent sodium current, *I*_Leak_ is the leak current, and $I_{\text{SynE}}^{i}$ and $I_{\text{SynI}}^{i}$ are the currents by excitatory synapses and inhibitory synapses, respectively. The ionic current *I*_NaP_ and leak current *I*_Leak_ are described as

(2) $$\begin{array}{l} I_{\text{Nap}}\left(V_{i},h_{i}\right)={\hat{g}}_{\text{Nap}}m_{\text{Nap}}\left(V_{i}\right)h_{i}\left\{V_{i}-{\text{E}}_{\text{Na}}\right\}\;i=\text{F},\text{E}\\ \;I_{\text{Leak}}\left(V_{i}\right)=\{\begin{array}{c} \;{\hat{g}}_{\text{Leak}}^{\text{RG}}\left\{V_{i}-{\text{E}}_{\text{Leak}}^{\text{RG}}\right\}\;i=\text{F},\text{E }\\ {\hat{g}}_{\text{Leak}}^{\text{InRG}}\left\{V_{i}-{\text{E}}_{\text{Leak}}^{\text{InRG}}\right\}\;i=\text{IF},\text{IE} \end{array} \end{array}$$

where ${\hat{\text{g}}}_{\text{Nap}}$, ${\hat{\text{g}}}_{\text{Leak}}^{\text{RG}}$, and ${\hat{\text{g}}}_{\text{Leak}}^{\text{InRG}}$ are the maximum conductances of the corresponding current, and E_Na_, ${\text{E}}_{\text{Leak}}^{\text{RG}}$, and ${\text{E}}_{\text{Leak}}^{\text{InRG}}$ are the reversal potentials of the corresponding current. In addition, *m*_Nap_ is the activation of the sodium channel of the RG neurons and is described as

(3) $$\begin{array}{r} m_{\text{Nap}}\left(V_{i}\right)=\frac{1}{1+\exp\left(-\frac{V_{i}+40.0}{6.0}\right)}\;i=\text{F},\text{E} \end{array}$$

The dynamics of the inactivation of the sodium channel *h*_*i*_ of the RG neurons (*i* = F, E) is given by

(4) $$\begin{array}{r} \tau \left(V_{i}\right){\dot{h}}_{i}=h_{\infty }\left(V_{i}\right)-h_{i}\;i=\text{F},\text{E} \end{array}$$

where

(5) $$\begin{array}{r} h_{\infty }\left(V_{i}\right)=\frac{1}{1+\exp\left(\frac{V_{i}+45.0}{4.0}\right)}\\ \;\tau \left(V_{i}\right)=320+\frac{320}{\cosh\left(\frac{V_{i}+35.0}{15.0}\right)}\text{ ms }i=\text{F},\text{E} \end{array}$$

The currents generated by the synapses $I_{\text{SynE}}^{i}$ and $I_{\text{SynI}}^{i}$ are given by

(6) $$\begin{array}{r} I_{\text{SynE}}^{i}\left(V\right)={\hat{\text{g}}}_{\text{SynE}}\left\{V_{i}-{\text{E}}_{\text{SynE}}\right\}\left\{\underset{j=\left\{\text{F},\text{E},\text{IF},\text{IE}\right\}}{\sum }{\text{a}}_{ij}f\left(V_{j}\right)+{\text{c}}_{i}d+{\text{w}}_{i}s_{i}\right\}\\ I_{\text{SynI}}^{i}\left(V\right)={\hat{\text{g}}}_{\text{SynI}}\left\{V_{i}-{\text{E}}_{\text{SynI}}\right\}\left\{\underset{j=\left\{\text{F},\text{E},\text{IF},\text{IE}\right\}}{\sum }{\text{b}}_{ij}f\left(V_{j}\right)\right\}\;i=\text{F},\text{E},\text{ IE},\text{IF} \end{array}$$

where ${\hat{\text{g}}}_{\text{SynE}}$ and ${\hat{\text{g}}}_{\text{SynI}}$ are the maximum conductances of the corresponding current, E_SynE_ and E_SynI_ are the reversal potentials of the corresponding current, *d* is the tonic drive from the supraspinal region, *s*_*i*_ (*i* = F, E, IF, IE) is the feedback input from sensory fibers, and a_*ij*_, b_*ij*_, c_*i*_, and w_*i*_ (*i, j* = F, E, IF, IE) are the weight coefficients. Moreover, the output function *f* translates *V* into the integrated population activity and is given by

(7) $$\begin{array}{r} f\left(V_{i}\right)=\{\begin{array}{cc} 0 & V_{i}<{\text{V}}_{\text{th}}\\ V_{i}-{\text{V}}_{\text{th}} & {\text{V}}_{\max\;}>V_{i}\geq {\text{V}}_{\text{th}}\\ 1 & V_{i}\geq {\text{V}}_{\max} \end{array}\;i=\text{F},\text{E},\text{IF},\text{IE} \end{array}$$

where V_th_ and V_max_ are the lower and upper threshold potentials, respectively. The differential equations of Equations (1), (2), and (4) were solved numerically using the fourth-order Runge-Kutta method with a step size of 0.01 ms. The parameter values are shown in Appendix A.

### Modeling the Effects of Phase-Dependent Afferent Stimulation

**Figure 2:**
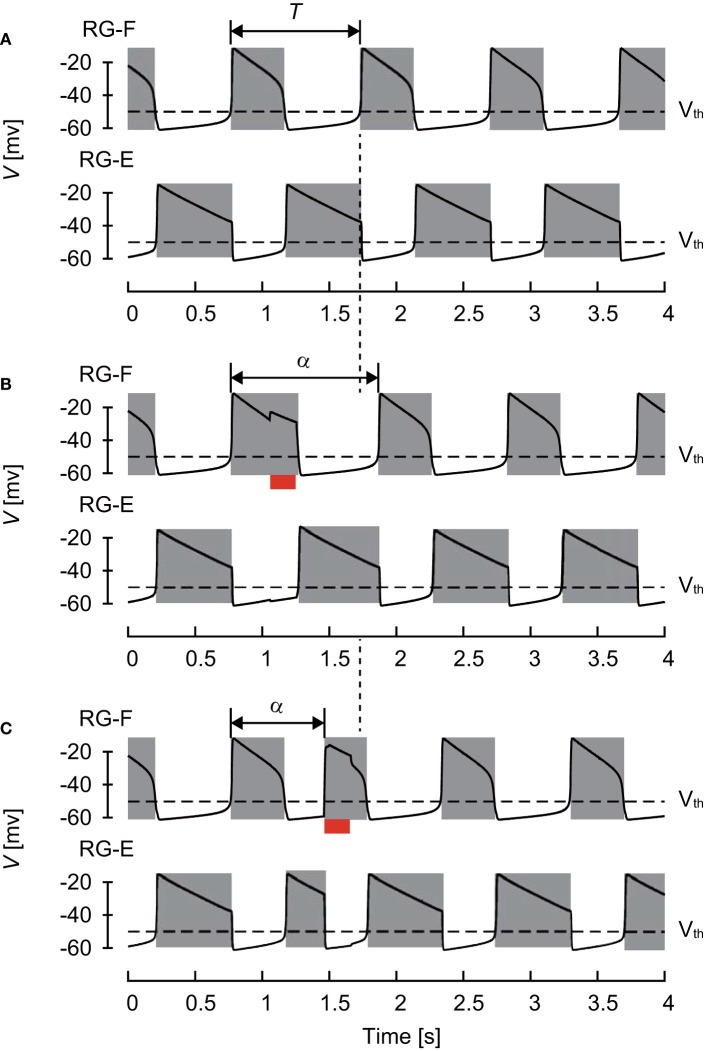
Changes of membrane potential of RG-F (top panel) and RG-E (bottom panel) neurons **(A)** without any stimulation, **(B)** with stimulation applied to the flexor side during flexor phase, and **(C)** with stimulation applied to the flexor side during extensor phase. The red bars indicate the application of stimulation. Gray regions indicate active phases. The applied stimulation increased the duration of the current flexor phase and cycle period in **(B)** and initiated the flexor phase and decreased the cycle period in **(C)**. Both stimulations produced phase shifts.

The CPG model produced rhythmic activity and exhibited stable oscillations, as shown in Figure 2. The active phase for each neuron was defined as the time interval during which the neuron's potential was higher than V_th_ and the silent phase as the time interval when the potential was lower than V_th_. The cycle period *T* was defined as the time interval between two consecutive onsets of the active phase. The phase of oscillation was defined as ϕ = 2π*t*/*T* ∈ [0, 2π).

The CPG also received external (“sensory”) signals (Figure 1). Based on a previous study (Demir et al., 1997), which investigated the response of a single neuron model to stimulations, we used depolarizing stimuli applied at different phases of oscillatory activity. Specifically, after oscillation stabilized, we applied a 200 ms stimulus to the flexor (RG-F and In-F) or extensor (RG-E and In-E) neurons. The intensity of stimulation was *s*_F_ = *s*_IF_ = 0.2 and *s*_E_ = *s*_IE_ = 0.0 for the flexor side and *s*_F_ = *s*_IF_ = 0.0 and *s*_E_ = *s*_IE_ = 0.2 for the extensor side in Equation (6). Suppose that the neuron activity is perturbed by the stimulation at phase ϕ_*s*_ ∈ [0, 2π) and the period changes from *T* to α(ϕ_*s*_), as shown in Figure 2. To show the phase shift of the neuron activity in response to the stimulation, we define

(8) $$\begin{array}{r} \Delta \left(\phi_{s}\right)=2\pi \frac{\alpha \left(\phi_{s}\right)-T}{T} \end{array}$$

### Calculation of Nullcline

The nullcline is a set of points at which the derivative of a differential equation is equal to zero. It reflects the structure of the solution of the differential equation. To investigate the mechanism of the phase-dependent response of the CPG model, we used a nullcline-based method. The state variable of the CPG model is given by (***V***, ***h***). The nullclines for the RG neurons are given by

(9) $$\begin{array}{ll} \begin{array}{l} {\text{N}}_{i}^{\text{V}}=\left\{\left(V,h\right)\;|\;{\dot{V}}_{i}=0\right\}\\ {\text{N}}_{i}^{\text{h}}=\left\{\left(V,h\right)\;|\;{\dot{h}}_{i}=0\right\} \end{array} & i=\text{ F},\text{ E} \end{array}$$

To clarify the dynamics of each RG neuron, we focused on the *V*_*i*_-*h*_*i*_ space (*i* = F, E) for the nullclines by assuming that the other variables *V*_*j*_ (*j* = F, E, IF, IE, *j*≠*i*) and *h*_*k*_ (*k* = F, E *k*≠*i*) are on the stable oscillation with phase ϕ. Therefore, we modify ${\text{N}}_{i}^{\text{V}}$ and ${\text{N}}_{i}^{\text{h}}$ in Equation (9) as

(10) $$\begin{array}{r} {\hat{\text{N}}}_{i}^{\text{V}}\left(\phi \right)=\left\{\left(V_{i},h_{i}\right)\;|\;{\dot{V}}_{i}=0,\;V_{j}=V_{j}\left(\phi \right),h_{k}=h_{k}\left(\phi \right)\right\}\\ {\hat{\text{N}}}_{i}^{\text{h}}\left(\phi \right)=\left\{\left(V_{i},h_{i}\right)\;|\;{\dot{h}}_{i}=0,\;V_{j}=V_{j}\left(\phi \right),h_{k}=h_{k}\left(\phi \right)\right\}\\ \;i=\text{F},\text{E }j=\text{F},\text{E},\text{IF},\text{IE }j\neq i\;k=\text{F},\text{ E }k\neq i \end{array}$$

For ${\hat{\text{N}}}_{i}^{\text{V}}\left(\phi \right)$ and ${\hat{\text{N}}}_{i}^{\text{h}}\left(\phi \right)$, we can write *h*_*i*_ = *h*_*i*_(*V*_*i*_; ϕ), as explained in Appendix B.

## Results

### Phase-Dependent Response

**Figure 3:**
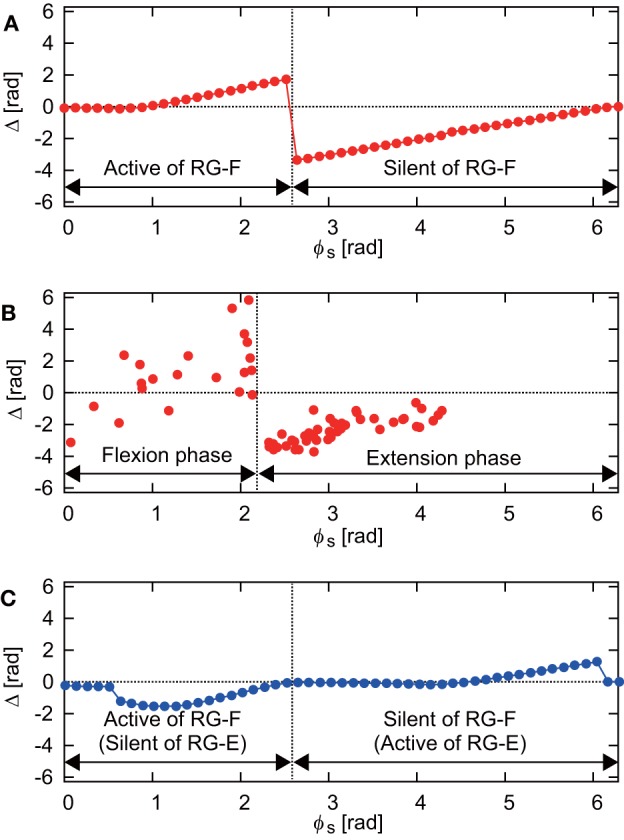
**(A)** Phase-dependent response of the RG-F neuron by stimulating sensory fibers of the flexor side. **(B)** Response against flexor muscle stimulation during fictive locomotion in cats (adapted from Schomburg et al., 1998). The flexion (extension) phase corresponds to the active (silent) phase of the RG-F neuron of the CPG model. **(C)** Phase-dependent response of the RG-F neuron by stimulating sensory inputs on the extensor side.

Figure 3A shows the phase shift Δ of the RG-F neuron activity after stimulation of sensory inputs on the flexor side at ϕ_*s*_. When stimulation was applied during the silent phase of RG-F (2.51 ≤ ϕ_*s*_ < 2π), it caused the transition to the active phase to occur earlier and this advanced start decreased with ϕ_*s*_. In contrast, almost no phase shift occurred when stimulation was applied at the beginning of the active phase of RG-F (0 ≤ ϕ_*s*_ < 1.00). However, the neuron activity was delayed by the stimulation during the middle and end of the active phase (1.00 ≤ ϕ_*s*_ < 2.51). These trends were similar to those observed during fictive locomotion in cats (Schomburg et al., 1998; Frigon et al., 2010), as shown in Figure 3B. Figure 3C shows Δ of the RG-F neuron activity by the stimulating sensory fibers of the extensor side. The active and silent phase of the RG-F neuron corresponds to the silent and active phase, respectively, of the RG-E neuron. The neuron activity was advanced at the middle of the silent phase of the RG-E neuron and was delayed at the end of the active phase of the RG-E neuron. The response of the stimulation of the extensor side was qualitatively similar to that of the flexor side. Moreover, these trends were similar to those seen in animal experiments (Schomburg et al., 1998; Frigon et al., 2010). The effects of the stimulation duration and intensity are further investigated in Figure S1 in Appendix C.

### Analysis on Nullclines

**Figure 4:**
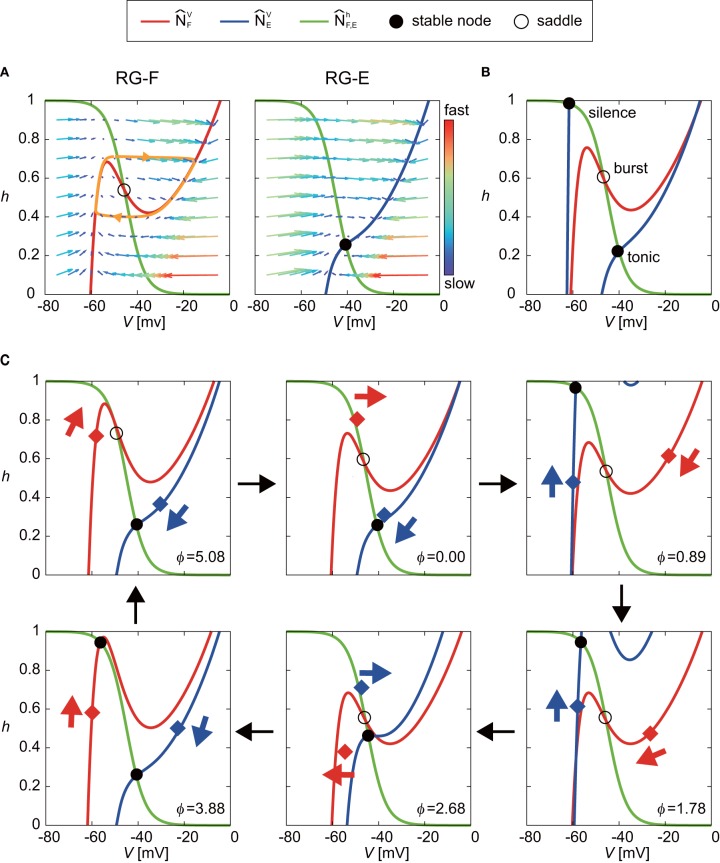
Roles of nullclines of RG-F and RG-E neurons to produce oscillatory behaviors. The green lines show ${\hat{\text{N}}}_{\text{F}}^{\text{h}}$ and ${\hat{\text{N}}}_{\text{E}}^{\text{h}}$. The red and blue lines show ${\hat{\text{N}}}_{\text{F}}^{\text{V}}$ and ${\hat{\text{N}}}_{\text{E}}^{\text{V}}$, respectively. Circles indicate intersections of nullclines [filled circles for both negative eigenvalues (stable node) and open circles for negative and positive eigenvalues (saddle)]. **(A)** ${\hat{\text{N}}}_{\text{F}}^{\text{V}}$ and ${\hat{\text{N}}}_{\text{E}}^{\text{V}}$ with the vector field for the case without synaptic connections from other neurons. The saddle produces a limit cycle (orange orbit) while stable node does not produce any oscillatory behavior. **(B)** Schematic illustration of changes in ${\hat{\text{N}}}_{\text{F}}^{\text{V}}$ and ${\hat{\text{N}}}_{\text{E}}^{\text{V}}$ induced by synaptic connections from other neurons. The intersection of ${\hat{\text{N}}}_{\text{F}}^{\text{V}}$ and ${\hat{\text{N}}}_{\text{F}}^{\text{h}}$ almost remains saddle (burst mode), which induces an oscillatory behavior. On the other hand, ${\hat{\text{N}}}_{\text{E}}^{\text{V}}$ transitions between two positions depending on the inhibitory signal from the contralateral side. This transition produces oscillatory behavior between tonic and silence modes for the extensor side. **(C)** Detailed illustration of our model at ϕ = 0, 0.89, 1.78, 2.68, 3.88, and 5.08 rad. Red and blue diamonds are (*V*_F_, *h*_F_) and (*V*_E_, *h*_E_), respectively, and these points move in accordance with eigenvalues, as indicated by arrows.

Even though the oscillatory behavior of the RG-E neuron was similar to that of the RG-F neuron as shown in Figure 2, the oscillating mechanism was different due to different nullclines as suggested in previous studies (Spardy et al., 2011a,b; Molkov et al., 2015). To understand this mechanism, we briefly explain the roles of nullclines in our neuron model. Figure 4A shows the nullclines ${\hat{\text{N}}}_{\text{F}}^{\text{V}}$, ${\hat{\text{N}}}_{\text{E}}^{\text{V}}$, ${\hat{\text{N}}}_{\text{F}}^{\text{h}}$, and ${\hat{\text{N}}}_{\text{E}}^{\text{h}}$ with the vector field for the case without synaptic connections from other neurons. While ${\hat{\text{N}}}_{\text{F}}^{\text{h}}$ and ${\hat{\text{N}}}_{\text{E}}^{\text{h}}$ are identical and have a sigmoid shape, ${\hat{\text{N}}}_{\text{F}}^{\text{V}}$ and ${\hat{\text{N}}}_{\text{E}}^{\text{V}}$ have different cubic curves. In particular, while ${\hat{\text{N}}}_{\text{F}}^{\text{V}}$ has two distinct inflection points and the sign of the slope changes at the inflection points, ${\hat{\text{N}}}_{\text{E}}^{\text{V}}$ changes monotonically. Because two eigenvalues at the intersection of ${\hat{\text{N}}}_{\text{F}}^{\text{V}}$ and ${\hat{\text{N}}}_{\text{F}}^{\text{h}}$ are positive and negative, the intersection is a saddle, which induces a limit cycle (orange orbit) due to the following three characteristics; (1) the trajectory approaches ${\hat{\text{N}}}_{\text{F}}^{\text{V}}$, especially its branches with positive slope due to the difference of the time constants between the dynamics of *V* and *h*, (2) the trajectory close to the positive branches moves along them until reaching the inflection points, and (3) the trajectory jumps to the opposite positive branch at the inflection points. In contrast to the case for the RG-F neuron, the two eigenvalues at the intersection of ${\hat{\text{N}}}_{\text{E}}^{\text{V}}$ and ${\hat{\text{N}}}_{\text{E}}^{\text{h}}$ are both negative and the intersection is stable node. The trajectory is attracted to this node and stays there as long as the node exists. Therefore, the RG-E neuron does not show any oscillatory behavior.

The synaptic connections from other neurons change ${\hat{\text{N}}}_{\text{F}}^{\text{V}}$ and ${\hat{\text{N}}}_{\text{E}}^{\text{V}}$ as shown schematically in Figure 4B, so that both RG-F and RG-E neurons show oscillatory behavior. On the one hand, although the intersection of ${\hat{\text{N}}}_{\text{F}}^{\text{V}}$ and ${\hat{\text{N}}}_{\text{F}}^{\text{h}}$ temporarily forms a stable node, it remains close to the saddle point (burst mode), which produces an oscillatory behavior. On the other hand, while the intersection of ${\hat{\text{N}}}_{\text{E}}^{\text{V}}$ and ${\hat{\text{N}}}_{\text{E}}^{\text{h}}$ remains stable, ${\hat{\text{N}}}_{\text{E}}^{\text{V}}$ transitions between two positions due to an inhibitory signal from the contralateral side, one of which has a high *V* at the intersection (tonic mode) and the other of which has a low *V* (silence mode). These transitions produce an oscillatory behavior. Figure 4C shows the details of our model at ϕ = 0, 0.89, 1.78, 2.68, 3.88, and 5.08 rad to show how the nullclines changed during one cycle.

### Shortening of Activity Duration During Silent Phase

**Figure 5:**
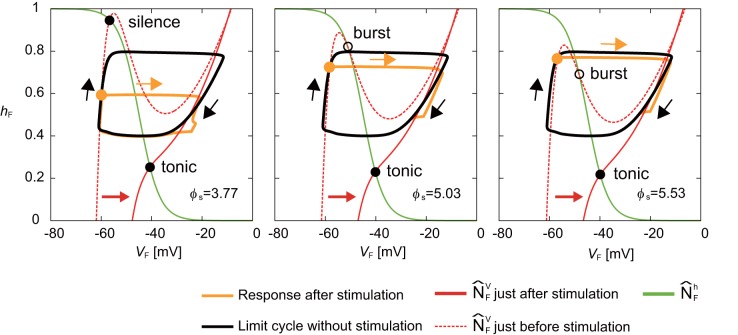
Response of a RG-F neuron on the *V*_*F*_-*h*_*F*_ plane by stimulating the flexor side at ϕ_*s*_ = 3.77, 5.03, and 5.53 rad. The black line shows the limit cycle without stimulation. Stimulation is applied at filled orange circles. Disturbed trajectories (orange line) take a shortcut to enter the limit cycle at different positions depending on ϕ_*s*_. Earlier ϕ_*s*_ has a larger truncated trajectory. The green line shows ${\hat{\text{N}}}_{\text{F}}^{\text{h}}$. The red dashed and solid lines show ${\hat{\text{N}}}_{\text{F}}^{\text{V}}$ just before and after the stimulation, respectively. The intersection of ${\hat{\text{N}}}_{\text{F}}^{\text{h}}$ and ${\hat{\text{N}}}_{\text{F}}^{\text{V}}$ changed from silence or burst mode to tonic mode by the stimulation.

Next, we investigated the mechanism for the phase-dependent response during the silent phase. Figure 5 shows the responses on the *V*_*F*_-*h*_*F*_ plane by the stimulation of the flexor side at ϕ_*s*_ = 3.77, 5.03, and 5.53 rad. The disturbed trajectories took a shortcut to the limit cycle at different positions depending on ϕ_*s*_, which decreased the activity duration and advanced the neural activity. As shown in Equations (1), (4), and (6), while stimulation directly influences the membrane potential *V*_*i*_ (*i* = F,E), it does not influence the inactivation of the sodium channel *h*_*i*_ (*i* = F,E). Therefore, a shortcut was produced in the direction of *V*_*i*_. Moreover, for the same reason, as ϕ_*s*_ occurs earlier, the shortcut has a larger truncated trajectory and the neural activity is more advanced. Although the intersection of ${\hat{\text{N}}}_{\text{F}}^{\text{h}}$ and ${\hat{\text{N}}}_{\text{F}}^{\text{V}}$ before the stimulation was in silence or burst mode, it suddenly changed to tonic mode after the stimulation, which attracted the trajectory toward the intersection and shortened the neuron activity.

**Figure 6:**
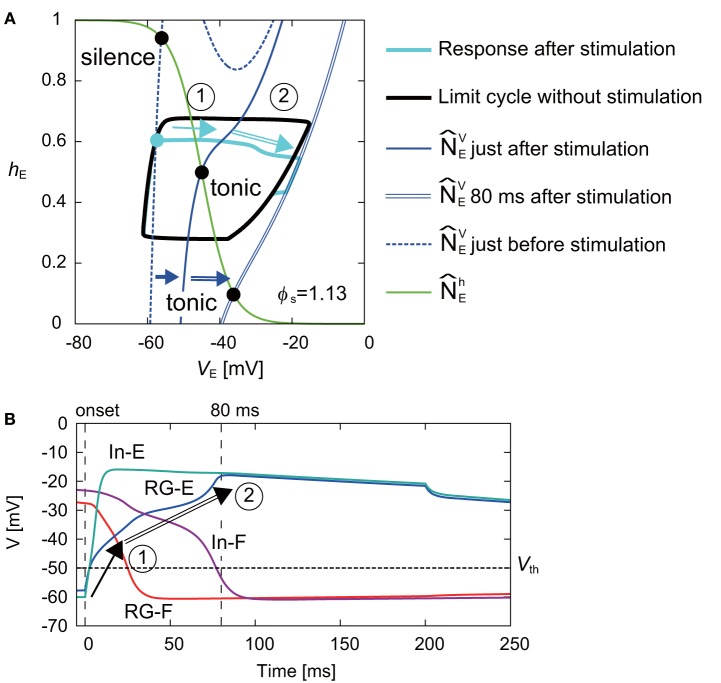
**(A)** Response of RG-E neuron on the *V*_E_-*h*_*E*_ plane by stimulating the extensor side at ϕ_*s*_ = 1.13 rad. The black line shows the limit cycle without stimulation. Stimulation was applied at filled cyan circle. The blue dashed and solid lines show ${\hat{\text{N}}}_{\text{E}}^{\text{V}}$ just before and after the stimulation, respectively. While ${\hat{\text{N}}}_{\text{E}}^{\text{V}}$ moved to the right just after the stimulation and the intersection with ${\hat{\text{N}}}_{\text{E}}^{\text{h}}$ (green line) became tonic mode, the disturbed trajectory (cyan line) moved to the right without entering the limit cycle (①). ${\hat{\text{N}}}_{\text{E}}^{\text{V}}$ gradually further moved to the right (blue double line shows ${\hat{\text{N}}}_{\text{E}}^{\text{V}}$ at 80 ms after the stimulation) and the trajectory was finally cut short to the limit cycle (②). **(B)** Time profiles of four neurons from the onset of the stimulation to the end of the shortcut. The vertical lines show the onset and 80 ms after the stimulation. The horizontal line shows V_th_. After the stimulation, the membrane potentials of the RG-E and In-E neurons rapidly changed and crossed over V_th_ (①). After that, while the membrane potentials of the RG-F and In-F neurons decreased due to the inhibitory signal from the In-E neuron and crossed over V_th_, the membrane potential of the RG-E neuron gradually increased. As a result, the decrease of the inhibitory signal from the flexor side increased the activity of the RG-E, which induced the shortcut (②).

Figure 6A shows the response on the *V*_E_-*h*_*E*_ plane by the stimulation of the extensor side at ϕ_*s*_ = 1.13 rad. While ${\hat{\text{N}}}_{\text{E}}^{\text{V}}$ moved to the right and the intersection of ${\hat{\text{N}}}_{\text{E}}^{\text{h}}$ and ${\hat{\text{N}}}_{\text{E}}^{\text{V}}$ changed from the silence to tonic mode just after the stimulation, the movement of ${\hat{\text{N}}}_{\text{E}}^{\text{V}}$ was smaller than that of ${\hat{\text{N}}}_{\text{F}}^{\text{V}}$ when the flexor side was stimulated (Figure 5). After the stimulation, although the disturbed trajectory moved to the right, it did not completely enter the limit cycle (① in Figure 6A). However, ${\hat{\text{N}}}_{\text{E}}^{\text{V}}$ gradually moved to the right and the intersection of ${\hat{\text{N}}}_{\text{E}}^{\text{h}}$ and ${\hat{\text{N}}}_{\text{E}}^{\text{V}}$ also further moved to the right. As a result, the trajectory eventually took a shortcut to the limit cycle (② in Figure 6A). Although the shortcut was induced by the change of the intersection of ${\hat{\text{N}}}_{\text{E}}^{\text{h}}$ and ${\hat{\text{N}}}_{\text{E}}^{\text{V}}$ from the silence to tonic mode in the same way as that of the stimulation of the flexor side (Figure 5), it was delayed due to an inhibitory signal from the flexor side just after the stimulation. More specifically, Figure 6B shows the time profiles of the neurons after the onset of the stimulation. Just after the stimulation, the membrane potentials (*V*_E_ and *V*_IE_) of the RG-E and In-E neurons increased immediately and crossed over V_th_ (① in Figure 6B), which changed the effect on the connected neurons described by Equation (7). The immediate change of the In-E neuron changed the activities of the other neurons. Especially, the membrane potentials (*V*_F_ and *V*_IF_) of the RG-F and In-F neurons decreased due to the inhibitory signal from the In-E neuron and crossed over V_th_. The decrease of the inhibitory signal from the flexor side increased *V*_E_ (② in Figure 6B), which induced the shortcut.

### Prolongation of Activity Duration During Active Phase

**Figure 7:**
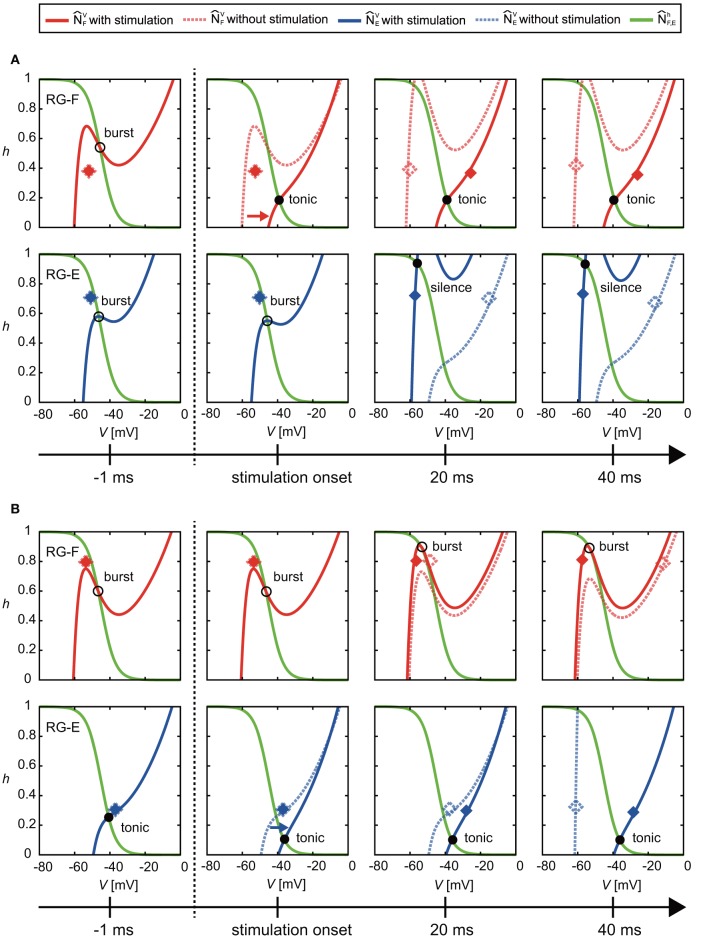
Change of ${\hat{\text{N}}}_{\text{F}}^{\text{V}}$ and ${\hat{\text{N}}}_{\text{E}}^{\text{V}}$ by stimulation at the end of the active phase of **(A)** the flexor side (ϕ_*s*_ = 2.39 rad) and **(B)** the extensor side (ϕ_*s*_ = 5.91 rad). Solid and dotted lines show the results of the cases with and without the stimulation, respectively. Red and blue diamonds mark the positions of (*V*_F_, *h*_F_) and (*V*_E_, *h*_*E*_), respectively (filled diamonds for stimulation and open diamonds for non-stimulation). In **(A)**, the stimulation moved ${\hat{\text{N}}}_{\text{F}}^{\text{V}}$ to the right and changed the intersection of ${\hat{\text{N}}}_{\text{F}}^{\text{h}}$ and ${\hat{\text{N}}}_{\text{F}}^{\text{V}}$ from burst to tonic mode. Furthermore, ${\hat{\text{N}}}_{\text{F}}^{\text{V}}$ showed almost no change for a while. These prolonged the activity duration. In addition, they kept the intersection of ${\hat{\text{N}}}_{\text{E}}^{\text{V}}$ and ${\hat{\text{N}}}_{\text{E}}^{\text{h}}$ silence mode for a while, which also delayed the neural activity. In **(B)**, the stimulation moved ${\hat{\text{N}}}_{\text{E}}^{\text{V}}$ to the right and maintained the intersection of ${\hat{\text{N}}}_{\text{F}}^{\text{h}}$ and ${\hat{\text{N}}}_{\text{F}}^{\text{V}}$ in tonic mode. These prolonged the activity duration of the RG-E neuron.

At the end of the active phase, the neural activity was delayed as shown in Figure 3. In the case without stimulation (*V*_F_, *h*_F_), of the RG-F neuron swooped down to the right inflection point of ${\hat{\text{N}}}_{\text{F}}^{\text{V}}$ at the end of the active phase, as shown in the panel for ϕ_*s*_ = 1.78 rad of Figure 4. However, the stimulation at the end of the active phase moved ${\hat{\text{N}}}_{\text{F}}^{\text{V}}$ to the right and changed the intersection of ${\hat{\text{N}}}_{\text{F}}^{\text{h}}$ and ${\hat{\text{N}}}_{\text{F}}^{\text{V}}$ from burst to tonic mode, as shown in Figure 7A. Furthermore, ${\hat{\text{N}}}_{\text{F}}^{\text{V}}$ showed almost no change for a while. These inhibited the deactivation of the RG-F neuron and prolonged the activity duration. In addition, the intersection of ${\hat{\text{N}}}_{\text{E}}^{\text{V}}$ and ${\hat{\text{N}}}_{\text{E}}^{\text{h}}$ changed from the burst to the silence mode and stayed in the silence mode for a while, which also delayed the neural activity. Figure 7B shows the case of the stimulation of the extensor side at the end of the active phase of the RG-E neuron. The RG-E neuron maintained the tonic mode due to the stimulation and this prolonged the activity duration. This response was similar to the case of flexor stimulation (Figure 7A).

## Discussion

In the present study, we investigated the underlying mechanism of the phase-dependent response of a half-center CPG model by applying a brief stimulation to it. The simulation results showed trends in the phase-dependent responses similar to those observed during fictive locomotion in cats (Schomburg et al., 1998; Frigon et al., 2010; Figures 3A,B).

It has been reported that the locomotor rhythm is reset to start a new flexion phase by an electrical stimulation to the flexor nerve in animals (Schomburg et al., 1998). Our simulation results suggest that, while the locomotor rhythm is reset to start a new flexion phase by stimulation during the silent phase, its start phase depends on the stimulation phase. The phase shifts of the RG-F neuron during the active phase (silent phase of the RG-E neuron) were also induced by stimulation of the extensor side (Figure 3C). However, in contrast to stimulation of the flexor side, the change in the intersection of the nullclines was smaller and formation of trajectory shortcut did not occur just after the stimulation of the extensor side (Figure 6). Instead, the In-E neuron was activated by the stimulation (we can estimate this using Equation S7 in Appendix D), which deactivated the RG-F and In-F neurons due to the inhibitory signal from the In-E neuron. As a result, the RG-E neuron was activated because of the deactivation of the neurons in the flexor side. These processes delayed the shortcut after the stimulation of the extensor side. Although the shortcut was delayed by the stimulation of the extensor side, the RG-E neuron had the potential to produce an immediate shortcut by stimulation, as in Figure 5, due to the nullcline intersection changing to a tonic mode when the stimulation intensity was larger as illustrated in Figure S2 in Appendix C.

At the end of the active phase, the neural activity was delayed by the stimulation. When the flexor side was stimulated, the intersection of the nullclines of the RG-F neuron changed from burst to tonic mode (Figure 7A). Similarly, the stimulation of the extensor side at the end of the active phase of the RG-E neuron prolonged the active phase by maintaining the tonic mode (Figure 7B). Even though the parameters of synaptic connection were different between the flexor and extensor sides, the mechanism of the active phase prolongation was the same (Figures 7A,B). As Figure S2 in Appendix C shows, the stimulation contributed to the nullcline intersection changing to a tonic mode irrespective of ϕ_*S*_. From our simulation results, the phase-dependency was caused by these acceleration and prolongation mechanisms, which were commonly induced by the change of the nullcline intersection to a tonic mode.

### Contribution of Different Afferent Types

Schomburg et al. (1998) demonstrated the resetting of the locomotor cycle in response to various flexor nerve stimulation during fictive locomotion. They employed both shorter stimulation trains (around 60 ms) at stimulation intensities activating joint and cutaneous afferents and longer stimulation trains (over 200 ms) at intensities activating only group I and II afferents. Other studies investigating the effects of sensory afferents on locomotor modulation also used relatively longer stimulation (for example, Ia and II afferents of extensor and flexor were stimulated for over 125 ms in Frigon et al., 2010; Ia or Ib afferents of extensor were stimulated for over 500 ms in Whelan et al., 1995; and II afferents of flexor were stimulated for over 200 ms in Perreault et al., 1995). Based on the conditions of these experiments, we used a stimulation lasting 200 ms. In addition, the effect of the stimulation intensity was also investigated in those experiments. Therefore, we examined the effect of the stimulation duration and intensity (Figures S1, S2 in Appendix C).

Functional roles of muscle spindles (Ia and II), Golgi tendon organs (Ib), and cutaneous afferent inputs during locomotion have been investigated in previous studies. During the stance phase, feedback from muscle spindles and Golgi tendon organs of extensor muscles prolong the duration of extensor activity (Guertin et al., 1995; Whelan et al., 1995) and muscle spindles in hip flexors contributed to initiation of the swing phase (Hiebert et al., 1996). At the beginning of the swing phase, stimulation of cutaneous nerves prolonged this phase (Duysens, 1977). As indicated above, the different responses depended on the locomotor phase. Yet, it remains unclear how the neural circuit of the CPG interacts with different types if sensory fibers and which neural circuits contributed to the generation of a phase-dependent response. In our present model, we did not identify the relative contributions of different afferent types to the CPG (Figure 1). Nevertheless, our model reproduced a phase-dependent response (Figure 3). Further experimental and computational studies are necessary to delineate anatomically and functionally plausible interactions between the CPG and the sensory afferents.

### Functional Roles of the Different Layers in CPGs

Although the anatomical structure of the CPG remains unclear, it has been suggested from modeling studies (Rybak et al., 2006a,b) that the CPG consists of a RG layer and a pattern formation (PF) layer. The PF layer is thought to determine the spatial motor pattern depending on the phase generated in the RG neurons; that is, it determines the distribution of the co-activated α-motoneurons over time. The muscle synergy hypothesis is one candidate for the determination of the distribution (Ivanenko et al., 2004, 2006) and modeling studies have shown that a motor control system based on this hypothesis could generate locomotion using musculoskeletal models (Aoi et al., 2010, 2013, 2019; Fujiki et al., 2018). In those models, the amplitudes of the α-motoneuron activities were determined in the PF layer. Based on this, it is suggested that the neurons in the PF layer modulate their amplitudes, which would be related to the phase-dependent response in terms of amplitude of the electromyography of Hoffmann-reflex during locomotion (Capaday and Stein, 1986; Yang and Stein, 1990). However, the neurons in the RG layer control the temporal aspect of the phase-dependent response as shown in the present study. As physiological experiments have shown, the feedback from muscle spindles contributed to the modulation of the muscle activity strength (Mayer et al., 2018) and the timing of the stance-to-swing and swing-to-stance transitions (Grillner and Rossignol, 1978; Hiebert et al., 1996; Akay et al., 2014). Therefore, the different layers of the CPG may explain the two different types of phase-dependency.

### Limitations of Model

In our study, we used the activity-based neuron model (Ermentrout, 1994; Markin et al., 2010; Molkov et al., 2015; Danner et al., 2016, 2017). This neuron model does not show spiking because it omits the potassium and fast-type sodium currents. Instead, this used a persistent sodium current, which enables the neuron model to generate bursting. Ausborn et al. (2018) showed that an activity-based neuron model preserved the principal dynamic features of neural activities as a half-center CPG. Even though our model did not include potassium and fast-type sodium currents, it reproduced the phase-dependent response and contributed to analysis of its dynamic structure.

### Interaction Between Body and Neural System During Adaptive Walking

In the present study, we focused on the phase-dependent response of the CPG activity during fictive locomotion. When animals walk, motor commands are sent to the leg muscles from the spinal CPG, and the CPG receives sensory signals from the leg nerves. While fictive locomotion is generated in an open-loop system, actual locomotion is generated in a closed-loop system. In addition to the analysis of fictive locomotion, in the future, we would like to investigate the entrainment mechanism through the dynamics of the CPG circuit, the body mechanical system, and the sensory system. Moreover, it has been suggested that the CPG consists of the RG and PF layers. While the RG layer determines the rhythm pattern of motor commands, the PF layer determines the spatial pattern (Rybak et al., 2006a). In the future, we would like to introduce the PF layer to our model to clarify further neural mechanisms of sensorimotor integration for adaptive locomotion.

## Data Availability Statement

The datasets generated for this study are available on request to the corresponding author.

## Author Contributions

SF and SA developed the study design in consultation with KT, SD, and DY. SF performed simulations and analyzed the data in consultation with SA, KT, and SD. SF, SA, SD, and IR wrote the manuscript. All authors reviewed and approved the manuscript.

### Conflict of Interest

The authors declare that the research was conducted in the absence of any commercial or financial relationships that could be construed as a potential conflict of interest.
