# Supplementary material for: Phase-Dependent Response to Afferent Stimulation During Fictive Locomotion: A Computational Modeling Study
Source: Front Neurosci. 2019 Nov 29;13:1288. doi: 10.3389/fnins.2019.01288 (PMC6896512; doi:10.3389/fnins.2019.01288)
Supplement: Supplementary file 1 [file Data_Sheet_1.pdf]

APPENDIX

A. Parameters of the neuron model

Table S1. Parameters of synaptic connections

	Target neuron			
Source	RG-F	RG-E	In-F	In-E
Excitatory connections a_{ij}				
RG-F	0	0	0.40	0
RG-E	0	0	0	0.40
Inhibitory connections b_{ij}				
In-F	0	0.7	0	0
In-E	0.10	0	0	0
Connections from supraspinal drive c_i	0.02	0.15	0	0
Afferent feedback connections w_i	1.0	1.0	1.0	1.0

We used the following values for the model parameters based on previous work (Molkov et al., 2015; Danner et al., 2016; Danner et al., 2017): $C = 20$ pF, $\hat{g}_{Nap} = 4.5$ nS, $\hat{g}_{Leak}^{RG} = 4.5$ nS, $\hat{g}_{Leak}^{InRG} = 2.8$ nS, $\hat{g}_{SynE} = 10$ nS, $\hat{g}_{SynI} = 10$ nS, $E_{Na} = 55$ mV, $E_{Leak}^{RG} = -62.5$ mV, $E_{Leak}^{RGIn} = -60$ mV, $E_{SynE} = -10$ mV, $E_{SynI} = -75$ mV, $V_{th} = -50$ mV, $V_{max} = 0$ mV, and $d = 1.0$. In addition, values for a_{ij}, b_{ij}, c_i, and w_i $(i, j = \mathrm{F, E, IF, IE})$ are shown in Table S1.

B. Calculation of the nullcline

For $\widehat{\mathrm{N}}_i^{\mathrm{V}}(\phi)$ $(i = \mathrm{F}, \mathrm{E})$ in Equation (10), from Equation (1), $\dot{V_i} = 0$ gives

$$0 = -I_{\mathrm{NaP}}(V_i, h_i) - I_{\mathrm{Leak}}(V_i) - I_{\mathrm{SynE}}^{i}(\boldsymbol{V}) - I_{\mathrm{SynI}}^{i}(\boldsymbol{V}) \qquad \text{(S1)}$$

where $V_j = V_j(\phi)$ $(j = \mathrm{F}, \mathrm{E}, \mathrm{IF}, \mathrm{IE},\ j \neq i)$. By substituting I_{NaP} in Equation (2) into Equation (S1), we obtain

$$\hat{\mathrm{g}}_{\mathrm{Nap}} m_{\mathrm{Nap}}(V_i) h_i \{V_i - \mathrm{E}_{\mathrm{Na}}\} = -I_{\mathrm{Leak}}(V_i) - I_{\mathrm{SynE}}^{i}(\boldsymbol{V}) - I_{\mathrm{SynI}}^{i}(\boldsymbol{V}) \qquad \text{(S2)}$$

From Equation (3), $m_{\mathrm{Nap}}(V_i) = 0$ when $V_i \to -\infty$. Because $\mathrm{E}_{\mathrm{Na}} = 55$ mV, and we focused on the range $-80\ \mathrm{mV} \leq V_i \leq 0$ mV, $m_{\mathrm{Nap}}(V_i)\{V_i - \mathrm{E}_{\mathrm{Na}}\} \neq 0$ was satisfied. Therefore, dividing Equation (S2) by $\hat{\mathrm{g}}_{\mathrm{Nap}} m_{\mathrm{Nap}}(V_i)\{V_i - \mathrm{E}_{\mathrm{Na}}\}$ gives

$$h_i = \frac{-I_{\mathrm{Leak}}(V_i) - I_{\mathrm{SynE}}^{i}(\boldsymbol{V}) - I_{\mathrm{SynI}}^{i}(\boldsymbol{V})}{\hat{\mathrm{g}}_{\mathrm{Nap}} m_{\mathrm{Nap}}(V_i)\{V_i - \mathrm{E}_{\mathrm{Na}}\}} = h_i(V_i; \phi) \qquad \text{(S3)}$$

For $\widehat{\mathrm{N}}_i^{\mathrm{h}}(\phi)$ $(i = \mathrm{F}, \mathrm{E})$ in Equation (10), from Equation (4), $\dot{h_i} = 0$ gives

$$0 = h_\infty(V_i) - h_i$$

Therefore, we obtain

$$h_i = h_\infty(V_i)$$

which does not depend on ϕ.

C. Effect of the stimulation duration and intensity

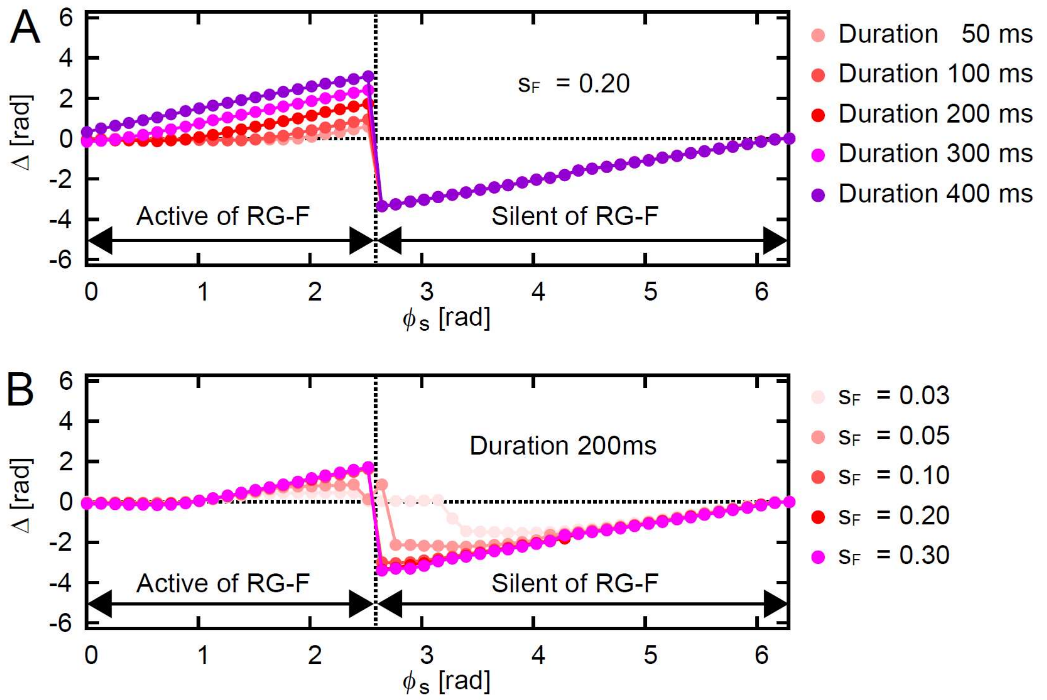

Figure S1. Phase-dependent response of the RG-F neuron by stimulating sensory fibers of the flexor side to investigate the effect of the stimulation (A) duration and (B) intensity. (A) The stimulation duration was varied (50, 100, 200, 300, and 400 ms) while the stimulation intensity was constant ($s_F = s_{IF} = 0.2$ and $s_E = s_{IE} = 0.0$). As the duration increased, the active phase became longer while the silent phase remained unchanged. (B) The stimulation intensity of the flexor side was varied ($s_F = s_{IF} = 0.03$, 0.05, 0.10, 0.20, and 0.30 and $s_E = s_{IE} = 0.0$) while the stimulation duration was constant (200 ms). As the intensity increased, the active phase became longer and the silent phase became shorter.

We investigated how the phase-dependent response changed depending on the stimulation duration and intensity. Figure S1A shows the phase-dependent response of the RG-F neuron to stimulation of sensory fibers on the flexor side, where we used the stimulation intensity $s_F = s_{IF} = 0.2$ and $s_E = s_{IE} = 0.0$ with various durations (50, 100, 200, 300, and 400 ms). The stimulation duration effected the prolongation of the active phase while it did not shorten the silent phase. Figure S1B shows the phase-dependent response of the RG-F neuron to stimulation of sensory fibers on the flexor side, where we used a stimulation duration of 200 ms and various intensities ($s_F = s_{IF} = 0.03$, 0.05, 0.10, 0.20, and 0.30 and $s_E = s_{IE} = 0.0$). Increased stimulation intensity prolonged the active phase and shortened the silent phase.

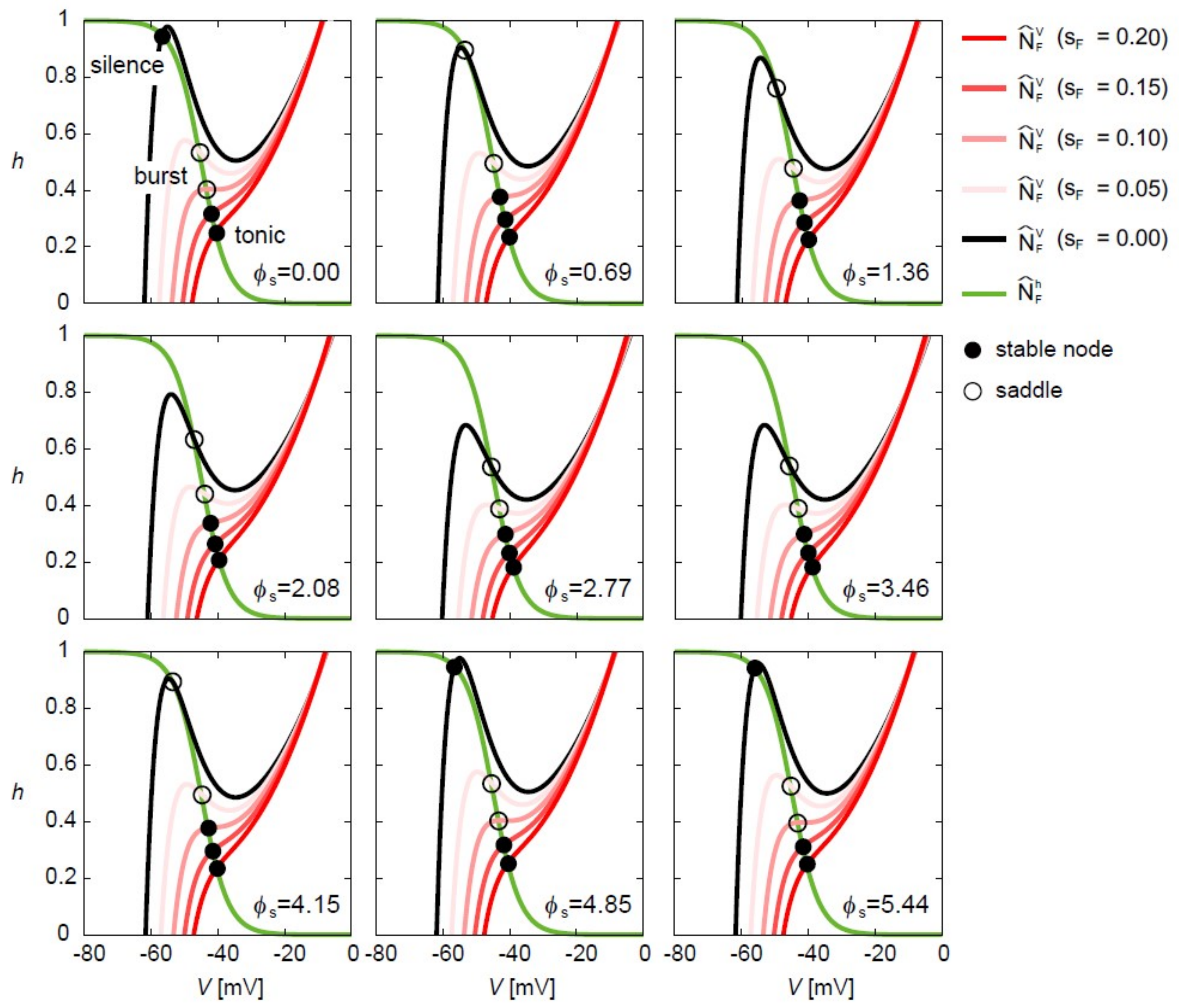

Figure S2. Change of $\widehat{\mathrm{N}}_{\mathrm{F}}^{\mathrm{V}}$ by different stimulation intensities of s_{F} at $\phi_{\mathrm{S}} = 0.00$, 0.69, 1.36, 2.08, 2.77, 3.46, 4.15, 4.85, and 5.44 rad. Circles indicate intersections of nullclines [filled circles for both negative eigenvalues (stable node) and open circles for negative and positive eigenvalues (saddle)]. When the intersection is saddle, that indicates the burst mode. A silence mode is indicated by an intersection at a stable node located higher than a saddle, and a tonic mode is indicated by an intersection at a stable node located lower than a saddle. Regardless of the stimulation phase, the intersection became tonic mode when the stimulation was large.

We also investigated how the nullclines changed depending on the stimulation intensity. Figure S2 compares $\widehat{\mathrm{N}}_{\mathrm{F}}^{\mathrm{V}}$ with synaptic connection between the cases of various stimulation intensities and values of ϕ_{S}. Regardless of the stimulation phase, the nullcline intersection became tonic mode when the stimulation was large.

D. Activation of In-E neuron by stimulation

The In-F and In-E neurons are represented by the one-dimensional differential equation given as Equation (1). The equation has an equilibrium point given by

$$0 = -I_{\mathrm{Leak}}(V_i) - I^{i}_{\mathrm{SynE}}(\boldsymbol{V}) - I^{i}_{\mathrm{SynI}}(\boldsymbol{V}) \tag{S4}$$

For the In-E neuron, Equation (S4) gives

$$V_{\mathrm{IE}} = \frac{\hat{\mathrm{g}}^{\mathrm{InRG}}_{\mathrm{Leak}}\mathrm{E}^{\mathrm{InRG}}_{\mathrm{Leak}} + \hat{\mathrm{g}}_{\mathrm{SynE}}\{\mathrm{a}_{\mathrm{IE,E}} f(V_{\mathrm{E}}) + \mathrm{w}_{\mathrm{IE}} s_{\mathrm{IE}}\}\mathrm{E}_{\mathrm{SynE}}}{\hat{\mathrm{g}}^{\mathrm{InRG}}_{\mathrm{Leak}} + \hat{\mathrm{g}}_{\mathrm{SynE}}\{\mathrm{a}_{\mathrm{IE,E}} f(V_{\mathrm{E}}) + \mathrm{w}_{\mathrm{IE}} s_{\mathrm{IE}}\}} \tag{S5}$$

During the silent phase of the RG-E neuron, $f(V_{\mathrm{E}}) = 0$ and, then Equation (S5) gives

$$V_{\mathrm{IE}} = \frac{\hat{\mathrm{g}}^{\mathrm{InRG}}_{\mathrm{Leak}}\mathrm{E}^{\mathrm{InRG}}_{\mathrm{Leak}} + \hat{\mathrm{g}}_{\mathrm{SynE}}\{\mathrm{w}_{\mathrm{IE}} s_{\mathrm{IE}}\}\mathrm{E}_{\mathrm{SynE}}}{\hat{\mathrm{g}}^{\mathrm{InRG}}_{\mathrm{Leak}} + \hat{\mathrm{g}}_{\mathrm{SynE}}\{\mathrm{w}_{\mathrm{IE}} s_{\mathrm{IE}}\}} \tag{S6}$$

When $V_{\mathrm{IE}} > \mathrm{V_{th}}$, the RG-F neuron is inhibited by the In-E neuron as shown in Equation (7). From Equation (S6), the condition is satisfied by

$$s_{\mathrm{IE}} > -\frac{\hat{\mathrm{g}}^{\mathrm{InRG}}_{\mathrm{Leak}}(\mathrm{E}^{\mathrm{InRG}}_{\mathrm{Leak}} - \mathrm{V_{th}})}{\hat{\mathrm{g}}_{\mathrm{SynE}}\mathrm{w}_{\mathrm{IE}}(\mathrm{E}_{\mathrm{SynE}} - \mathrm{V_{th}})} \tag{S7}$$

This indicates that $V_{\mathrm{IE}} > \mathrm{V_{th}}$ is satisfied when $s_{\mathrm{IE}} > 0.07$ and the parameter values given in the Appendix A are used.
